# Macronutrient intake during pregnancy in women with a history of obesity or gestational diabetes and offspring adiposity at 5 years of age

**DOI:** 10.1038/s41366-021-00762-0

**Published:** 2021-02-08

**Authors:** Jelena Meinilä, Miira M. Klemetti, Emilia Huvinen, Elina Engberg, Sture Andersson, Beata Stach-Lempinen, Saila Koivusalo

**Affiliations:** 1 Department of Food and Nutrition, University of Helsinki, Helsinki, Finland; 2 Department of Obstetrics and Gynecology, University of Helsinki and Helsinki University Hospital, Helsinki, Finland; 3 Department of Obstetrics and Gynecology, South Karelia Central Hospital, Lappeenranta, Finland; 4 Department of Medical and Clinical Genetics, University of Helsinki and Helsinki University Hospital, Helsinki, Finland; 5 Lunenfeld-Tanenbaum Research Institute, Mount Sinai Hospital, Toronto, ON Canada; 6 Folkhälsan Research Center, Helsinki, Finland; 7 Faculty of Medicine, University of Helsinki, Helsinki, Finland; 8 Children’s Hospital, Pediatric Research Center, University of Helsinki and Helsinki University Hospital, Helsinki, Finland

**Keywords:** Risk factors, Nutrition

## Abstract

**Background/objectives:**

The impact of maternal macronutrient intake during pregnancy on offspring childhood adiposity is unclear. We assessed the associations between maternal macronutrient intake during and after pregnancy with offspring adiposity at 5 years of age. Additionally, we investigated whether gestational diabetes (GDM), BMI, or breastfeeding modified these associations.

**Subjects/methods:**

Altogether, 301 mother–child dyads with maternal prepregnancy BMI ≥ 30 and/or previous GDM participated in the Finnish Gestational Diabetes Prevention Study (RADIEL) and its 5 years follow-up. Macronutrient intakes (E%) were calculated from 3-day food records collected at 5–18 weeks’ gestation, in the third trimester, and at 12 months and 5 years after pregnancy. Offspring body fat mass (BFM) and fat percentage (BF%) at 5 years were measured by bioimpedance. Statistical analyses were multivariate linear regression.

**Results:**

Mean (SD) prepregnancy BMI was 33(4) kg/m^2^. GDM was diagnosed in 47%. In normoglycemic women, higher first half of pregnancy n-3 PUFA intake was associated with lower offspring BFM (g) (*ß* −0.90; 95% CI −1.62, −0.18) and BF% (*ß* −3.45; 95% CI −6.17, −0.72). In women with GDM, higher first half of pregnancy n-3 PUFA intake was associated with higher offspring BFM (*ß* 0.94; 95% CI 0.14, 1.75) and BF% (*ß* 3.21; 95% CI 0.43, 5.99). Higher SFA intake in the third trimester and cumulative intake across pregnancy (mean of the first half and late pregnancy) was associated with higher BFM and BF% (across pregnancy: *ß* 0.12; 95% CI 0.03, 0.20 and *ß* 0.44; 95% CI 0.15, 0.73, respectively). Higher carbohydrate intake across pregnancy was associated with lower BFM (*ß* −0.044; 95% CI −0.086, −0.003), and borderline associated with BF% (*ß* −0.15; 95% CI −0.31, 0.00).

**Conclusions:**

The macronutrient composition of maternal diet during pregnancy is associated with offspring BFM and BF% at 5 years. GDM modifies the association between prenatal n-3 PUFA intake and offspring anthropometrics.

## Introduction

Obesity is rapidly increasing in children and adolescents worldwide. In 2019, 38 million children younger than 5 years were overweight or obese [1]. In accordance with global trends, over 25% of Finnish preschool-aged boys and over 15% of girls are currently overweight or obese [2]. Child obesity is associated with reduced quality of life in childhood [3], cardiometabolic morbidity in later life [4], and premature death [5]. Therefore, identification and targeting of modifiable risk factors for childhood obesity should be a public health priority.

Mounting evidence indicates that maternal health behaviors such as physical activity, smoking, and diet during pregnancy are associated with long-term health outcomes in the offspring [6–8]. Data from animal and human studies suggest that, in addition to maternal gestational diabetes (GDM) and obesity [9–11], in utero exposure to poor quality diet (e.g., under- or over-nutrition) predisposes the offspring to excess adiposity [12, 13]. Recent findings also suggest that the macronutrient composition of maternal diet during pregnancy is associated with indicators of newborn adiposity (body mass index (BMI), waist circumference, body fat mass (BFM), and body fat percentage (BF%)) [8, 14, 15]. However, only a few studies have examined the associations between maternal macronutrient intake and offspring adiposity at later ages, and analyses extending beyond maternal polyunsaturated fatty acid (PUFA) intake are particularly scarce [8, 16–19]. Some of the available studies have relied only on indirect measures of adiposity, such as weight [16] or BMI [17, 19]. Furthermore, only one study has attempted to differentiate between the effects of maternal nutrient intake during pregnancy and the child’s own nutrient intake in early childhood by analyzing maternal macronutrient intake after pregnancy as an indicator of the food environment of the offspring [16]. None of the existing studies have taken into account the child’s own macronutrient intake. Moreover, whether the impact of maternal macronutrient intake on the offspring body composition varies across gestation remains unclear.

The aim of this study was to examine the associations between the macronutrient composition of maternal diet during the first half of and late pregnancy and offspring anthropometric indicators at 5 years of age (age- and sex-adjusted BMI (ISO-BMI), waist-to-height ratio (WHtR), BFM, and BF%). To ensure that none of the observed associations between macronutrient intakes during pregnancy and offspring anthropometric indicators result from confounding by the child’s own food environment during childhood, we also studied the associations between maternal macronutrient intakes at 12 months and 5 years after pregnancy and offspring adiposity.

## Subjects and methods

All background and pregnancy data were originally collected during the RADIEL study, a multi-center randomized controlled intervention trial aimed at prevention of GDM with diet and physical activity counseling [20]. The trial involved a total of 787 women at high risk for GDM who delivered in the Helsinki metropolitan area or in Lappeenranta, Finland, during 2008–2011. The recruited women were obese (BMI ≥ 30 kg/m^2^) and/or had been diagnosed with GDM in a previous pregnancy. At enrollment, the women were either ≤20 weeks pregnant or planning pregnancy. Exclusion criteria were age <18 years, preexisting type 1 or 2 diabetes mellitus, physical disability, multiple pregnancy, severe psychiatric disorders, current substance abuse, substantial communication difficulties and using medication affecting glucose metabolism (e.g., corticosteroids, metformin, antidepressants with potential effects on glucose homeostasis).

**Fig. 1:**
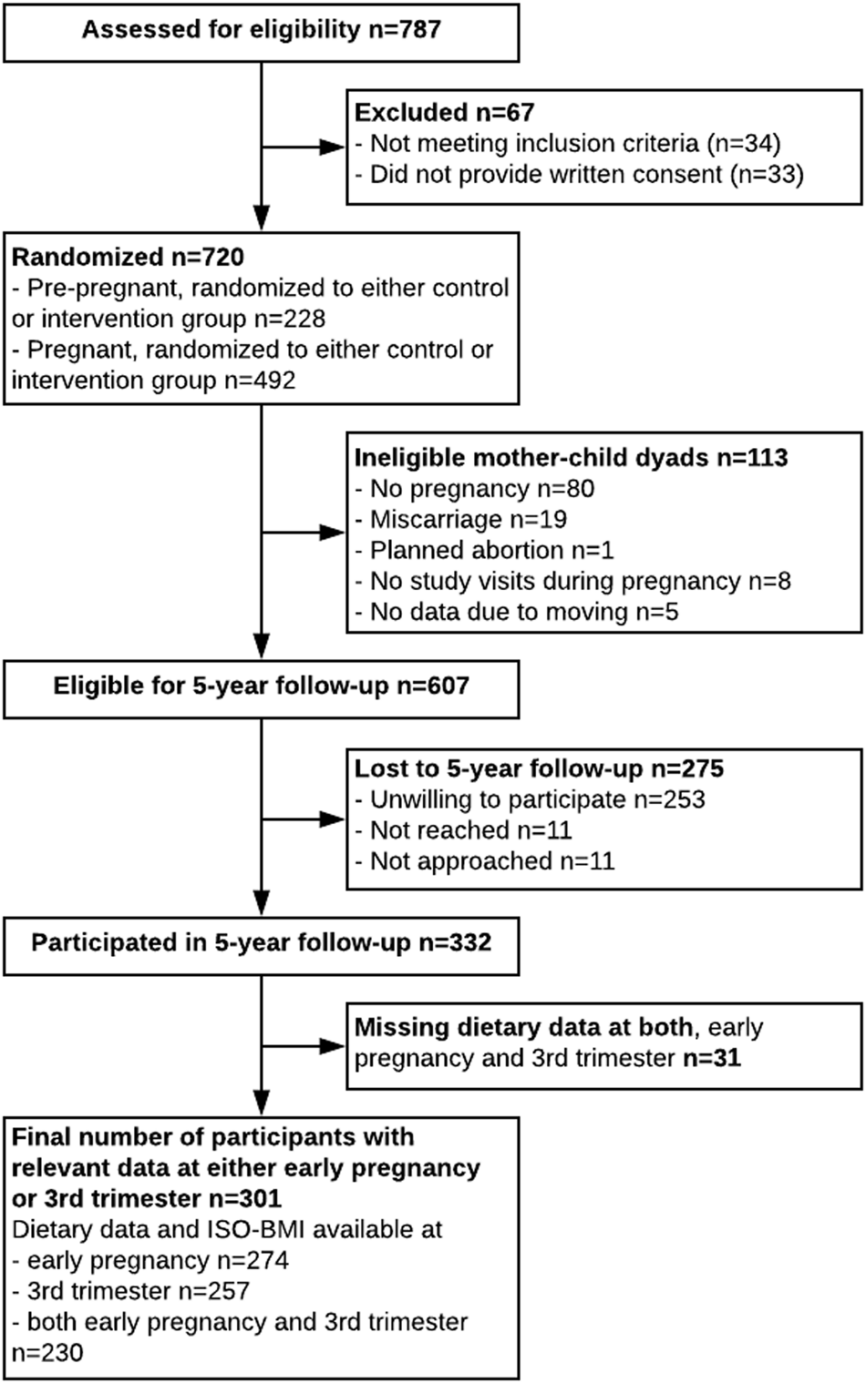
Formation of the study population. The first half of pregnancy contains gestational weeks from 5 to 18. ISO-BMI represents age- and sex-standardized BMI converted to adult scale and was calculated according to Saari et al. [23].

Five years after the trial, during 2013–2017, the participants and their offspring were invited for follow-up examinations. The study population for the present study was formed as depicted in Fig. 1. The number of mother–child pairs who participated in the follow-up was 332; 301 provided both a completed maternal 3-day food record at least once during pregnancy (in the first half of pregnancy 5–18 weeks’ gestation or in the third trimester) and the offspring’s weight and height at 5 years. At least one maternal food record and the offspring’s BFM and BF% measurements at 5 years of age were available from 274 pairs. Maternal 3-day food records from both the first half of pregnancy and third trimester of pregnancy and offspring’s weight and height at 5 years of age were available from 230 mother–child pairs, with offspring BFM and BF% at 5 years additionally available from 201 of these pairs. In the present study, the intervention and control groups were combined and treated as a cohort and observational analyses were performed. The study was conducted according to the guidelines laid down in the Helsinki Declaration of 1975 as revised in 1983. All procedures involving human subjects were approved by the Ethics Committee of the Department of Obstetrics and Gynecology of Helsinki and Uusimaa Hospital District. Written informed consent was obtained from all subjects in the main trial and in the 5-year follow-up. In the follow-up study, informed consent on behalf of the child was obtained from the parents.

### Nutrient intake of the mother and child

Maternal macronutrient intake during pregnancy was calculated from two 3-day food records collected in the first half of pregnancy (gestational weeks 5–18, mean 13 ± 2) and in the third trimester of pregnancy (mean gestational weeks 35 ± 1), as well as at 12 months and 5 years after delivery. At the initial trial enrollment visit in the first half of pregnancy, mothers received written instructions for completing food records on three consecutive days (two weekdays and a weekend day) immediately or as soon as possible after each meal. They were asked to report food and beverage labels and brand names as accurately as possible, and record amounts as household measures (e.g., tablespoon, plate, scoop) or in weight units, if available. Identical instructions were given for completing 3-day food records in the third trimester of pregnancy and at 12 months and 5 years after delivery.

Similarly, the offspring’s macronutrient intake at 5 years of age was calculated from 3-day food records completed by their caretaker (usually mother or father) with similar accuracy as instructed for maternal food records. If the 3-day recording period included daycare time, the childcare providers were asked to complete a separate food record with identical instructions. Recipes and ingredients used in daycare meals were collected from the food suppliers or the daycare kitchen.

Trained nutritionists processed and entered the food record data into a nutritional calculation software, AivoDiet (versions 2.0.1.5 and 2.2.0.1, Aivo Finland Oy, Turku, Finland). A table of usual portion sizes helped in converting household measures and volumes into grams [21]. The food composition database was provided by the Finnish National Institute for Health and Welfare (www.fineli.fi). The nutrient values in the database rely mostly on Finnish studies, in addition to data obtained from Finnish food industries and international food composition tables. The database includes standard recipes that are based on contemporary Finnish cookbooks. If a food or recipe reported in the food record was missing, a new recipe was created based on the food record.

### Collection of maternal background information

At the initial trial enrollment and 5-year follow-up visits, the mothers completed a background questionnaire on socio-demographic factors, earlier pregnancies and deliveries, and health-related behaviors during the preceding 6 months. Information on GDM was based on medical records and physician confirmation, except for two participants for whom this was unavailable. Prepregnancy BMI was calculated from weight and height measured at the first study visit (in those recruited before pregnancy) or was self-reported (in those recruited in the first half of pregnancy). Duration of breastfeeding (<4 months/≥4 months) was collected from the records of the child health centres which monitored the children’s health, growth, and development.

### Collection of offspring background data and anthropometric measurements

Data on offspring sex, birthweight, and gestational age at birth were collected from hospital records. Relative birthweight was calculated using Finnish fetal growth curves adjusted for sex and gestational age [22]. Measurements from the child participants were taken in the research centers of the Hospital District of Helsinki and Uusimaa or in South Karelia Central Hospital by trained study nurses. Height was measured with feet shoeless, flat, together, and against the wall. Weight was measured with a digital scale in light clothing. Waist circumference was measured at midway between the lowest ribs and the iliac crest. Measures of height, weight, and waist were rounded to one decimal. Offspring BFM and BF% at 5 years were measured during the 5-year visit using Bioelectrical Impedance Analysis (InBody 720 eight-polar tactile electrode system (Biospace Co., Ltd, Seoul, South Korea)). Offspring sex-specific BMI-for-age at 5 years was calculated according to the Finnish growth curves and was converted to adult scale (ISO-BMI) according to Saari et al. [23]. Offspring WHtR was calculated as waist circumference (cm) divided by height (m).

### Statistical methods

Descriptive characteristics are presented as means (SD) or frequencies (%). Macronutrient intakes in the first half of pregnancy and in the third trimester of pregnancy were analyzed both separately and combined (mean of the two measures) to represent cumulative intake across pregnancy. Associations between maternal macronutrient intakes and offspring *z*-birthweight, and ISO-BMI, WHtR, BFM, and BF% at 5 years were analyzed by linear regression analysis. Model 1 included mother’s energy intake as a covariate. Model 2 additionally included potential confounding factors such as maternal age during pregnancy, educational attainment (years), smoking in the first half of pregnancy (yes/no), GDM status (yes/no), intervention allocation (control/intervention during the main trial), maternal prepregnancy BMI, offspring sex, offspring age at 5-year follow-up visit, offspring’s *z*-birthweight, and offspring’s intake of the given nutrient (E%) at 5 years of age. Additionally, we constructed a model without the variables that could potentially be involved in the causal pathways between maternal macronutrient intake and offspring anthropometric measures: GDM status, intervention allocation, maternal prepregnancy BMI, offspring *z*-birthweight, and offspring’s intake of the index nutrient at 5 years of age. The results of this additional model are not presented since they did not differ substantially from those obtained with Model 2. Information on maternal leisure-time physical activity (LTPA) and duration of breastfeeding, potential confounders in the association between maternal diet and offspring anthropometric indicators, were missing from several participants (LTPA: 34 mothers with only first half of pregnancy food records, 17 mothers with only third trimester food records, and 42 mothers with two food records during pregnancy; breastfeeding: 28 mothers with only first half of pregnancy food records, 7 mothers with only third trimester food records, and 23 mothers with two food records during pregnancy). Therefore, these variables were omitted from the main analyses. However, in sensitivity analyses, LTPA and duration of breastfeeding were added separately as covariates, in addition to the variables of Model 2. We also tested nutrient interaction with variables GDM, duration of breastfeeding, and prepregnancy BMI on offspring anthropometric indicators. Each interaction was tested in a separate analysis by including in the models both the interaction term ([GDM × nutrient intake (E%)]/[duration of breastfeeding × nutrient intake (E%)]/[prepregnancy BMI × nutrient intake (E%)]) and its components as independent variables, together with the other covariates of Model 2. This was performed for all studied nutrients in all timepoints (first half of pregnancy, third trimester, and 12 months and 5 years after pregnancy). If an interaction was found, the analyses were performed in subgroups according to the variable with which the interaction was identified.

If an association between maternal intake of a macronutrient and offspring ISO-BMI, WHtR, BFM, or BF% was found, additional analyses were performed to assess whether the observed association was due to intake during pregnancy or due to maintained nutrient intake after pregnancy (which could reflect the food environment of the offspring). The nutrient intakes of Model 2 were replaced with maternal intakes at 12 months or 5 years after delivery, and the offspring’s own intakes of specific nutrients were excluded from the model.

We also tested quadratic associations between macronutrients and BFM and BF% by adding a squared macronutrient intake in addition to macronutrient intake in Model 2. These were tested in the same groups as the main analyses (i.e., in the total cohort, or in subgroups, if interactions were observed).

In case of violation of the assumption of normality, a bootstrap method was used. Equality of variances were tested by Levene’s test. Statistical software Stata, version 13.1 (Stata Corporation, College Station, TX, USA) was used in all analyses.

## Results

### Maternal characteristics

Maternal background characteristics are shown in Table 1. The mean (SD) maternal age was 33 (4) years and the mean prepregnancy BMI was 31 (6) kg/m^2^. Almost half of the mothers (*n* = 141; 47%) had GDM during the index pregnancy. The mean (SD) age of the offspring at follow-up was 5.0 years (0.5) and 138 (46%) were girls. The mean (SD) BF% of girls was 19 (6) and that of boys 15 (6). The mean (SD) ISO-BMI for both the girls and boys was 23 (4). Table 2 displays the mean maternal macronutrient intakes and time spent on LTPA during pregnancy, separately in the first half of pregnancy and third trimester, and combined to reflect total exposure across pregnancy.

**Table 1:** Maternal and offspring characteristics and offspring anthropometric indicators at 5 years of age in mother–child dyads with maternal 3-day food records completed in both first half of pregnancy (5–18 weeks’ gestation) and third trimester, and in those with food records completed only once during pregnancy.

	Food diary from 1st half of pregnancy and 3rd trimester (*n* = 230)		Missing values	Food diary from 1st half of pregnancy and/or 3rd trimester (*n* = 301)		Missing values
Mother						
Age, year, mean (SD)	32.9	(4.4)	0	33.0	(4.5)	0
BMI at 1st half of pregnancy, kg/m^2^, mean (SD)	30.9	(5.6)	0	31.1	(5.7)	0
Educational attainment, year, mean (SD)	14.7	(2.0)	0	14.6	(2.0)	1
Current smokers (in 1st trimester), *n* (%)	8	(4)	1	12	(4)	1
Gestational age at early pregnancy food recording, mean (SD)	13.1	(1.8)	1	13.1	(1.8)	1
Gestational age at 3rd trimester food recording, mean (SD)	34.9	(1.1)	2	34.9	(1.2)	4
Gestational diabetes, *n* (%)	101	(44)	0	141	(47)	0
Gestational age at delivery, week, mean (SD)	39.9	(1.4)	0	39.9	(1.5)	0
Breastfeeding, months, mean (SD)	4.2	(2.9)	17	4.2	(2.9)	21
Child						
Boys, *n* (%)	122	(53)	0	163	(54)	0
Girls	108	(47)	0	138	(46)	0
At birth						
Birthweight, g, mean (SD)	3632	(494)	0	3664	(503)	0
*z*-birthweight, mean (SD)	0.1	(0.9)	0	0.2	(1.0)	0
At 5-year follow-up						
Age, year, mean (SD)	5.0	(0.5)	0	5.0	(0.5)	0
Waist						
Boys	54.3	(3.5)	6	54.6	(3.9)	10
Girls	54.9	(5.0)	5	54.9	(4.9)	9
Waist–height ratio, mean (SD)						
Boys	48.7	(2.6)	6	48.8	(2.9)	10
Girls	49.8	(3.9)	5	49.7	(3.9)	9
BMI, mean (SD)						
Boys	16.0	(1.3)	0	16.1	(1.4)	0
Girls	16.4	(1.8)	0	16.4	(1.7)	0
ISO-BMI, mean (SD)						
Boys	22.9	(3.6)	0	23.1	(4.0)	0
Girls	22.5	(4.1)	0	22.6	(4.0)	0
Fat mass, kg, mean (SD)						
Boys	3.0	(1.3)	13	3.1	(1.5)	19
Girls	4.0	(2.0)	16	4.0	(4.0)	16
Fat as % from total bodyweight, mean (SD)						
Boys	14.6	(5.4)	13	14.9	(5.6)	19
Girls	19.2	(6.1)	29	19.1	(6.1)	16

*z*-birthweight represents sex- and gestational age-adjusted birthweight [22]. ISO-BMI represents age- and sex-standardized BMI converted to adult scale [23].

**Table 2:** Maternal macronutrient intakes and duration of leisure-time physical activity per week in first half of pregnancy, in the third trimester of pregnancy, and as an average of the two timepoints.

	1st half of pregnancy (gestational weeks 5–18)			3rd trimester of pregnancy				Combined 1st half and 3rd trimester		
	*n*	Mean	sd	*n*	Mean	sd	*p*	*n*	Mean	sd
Nutrition	274			257				230		
Energy intake, kcal/d		1900	420		1950	440	0.50		1930	380
Carbohydrates, E%		44.8	6.0		43.5	7.0	0.01		44.0	5.5
Sucrose, E%		8.5	3.8		8.5	4.5	0.31		8.4	3.3
Dietary fiber, g/1000 kcal		12.6	3.8		12.5	3.9	0.77		12.5	3.3
Total fat, E%		33.6	5.7		34.8	6.8	0.02		34.3	5.3
SFA, E%		12.3	2.9		12.8	3.5	0.10		12.6	2.7
MUFA, E%		11.6	2.3		11.9	2.6	0.04		11.8	2.0
n-3 PUFA, E%		1.8	0.4		1.8	0.5	0.50		1.8	0.3
n-6 PUFA, E%		4.5	1.1		4.7	1.4	0.03		4.6	1.0
Protein, E%		18.1	3.1		18.3	3.1	0.21		18.2	2.5
Duration of LTPA per week (min)	240	105	110	240	81	107	0.12	191	94	88

*E%* percentage of energy from total energy intake, *SFA* saturated fatty acids, *MUFA* monounsaturated fatty acids, *PUFA* polyunsaturated fatty acids, *LTPA* leisure-time physical activity.

### Maternal macronutrient intake and offspring relative birthweight

None of the maternal macronutrient intakes were associated with offspring’s relative birthweight (Supplementary Table 2).

### Maternal macronutrient intake and offspring ISO-BMI and waist-to-height ratio (WHtR)

GDM, BMI, or breastfeeding did not modify any associations between macronutrients and ISO-BMI and WHtR (all *p* for interaction >0.05). Higher third trimester saturated fatty acid (SFA) intake was associated with higher ISO-BMI (Model 2), but the first half of pregnancy intake or combined intake across pregnancy were not (Table 3). No associations between maternal macronutrient intakes (in the first half of pregnancy, third trimester, or combined) and WHtR were detected.

**Table 3:** Estimated change in offspring ISO-BMI and waist–height ratio (cm/m) associated with 1% isocaloric increase in specific macronutrient intake (except for fiber 1 g increase/1000 kcal) during pregnancy offset by concomitant drop in other nutrients (total energy is held constant).

	ISO-BMI										Waist–height ratio									
	Model 1					Model 2					Model 1					Model 2				
	*n*	*ß*	95% CI		*p*	*n*	*ß*	95% CI		*p*	*n*	*ß*	95% CI		*p*	*n*	*ß*	95% CI		*p*
Total fat																				
1st half of pregnancy	274	0.02	−0.07	0.11	0.66	263	0.01	−0.07	0.09	0.83	258	0.01	−0.07	0.08	0.86	249	0.01	−0.06	0.09	0.70
3rd trimester	257	0.04	−0.04	0.12	0.33	247	0.05	−0.01	0.12	0.12	243	0.03	−0.04	0.11	0.38	234	0.04	−0.03	0.10	0.30
Combined 1st half of pregnancy and 3rd trimester	230	0.02	−0.09	0.14	0.67	222	0.05	−0.05	0.14	0.36	219	0.01	−0.10	0.12	0.85	212	0.03	−0.07	0.12	0.58
SFA																				
1st half of pregnancy	274	0.09	−0.08	0.26	0.32	263	0.08	−0.08	0.24	0.32	258	0.08	−0.07	0.23	0.37	249	0.10	−0.04	0.24	0.17
3rd trimester	257	0.13	−0.01	0.27	0.08	247	0.13	0.01	0.25	0.035	243	0.12	−0.02	0.27	0.10	234	0.11	−0.02	0.24	0.11
Combined 1st half of pregnancy and 3rd trimester	230	0.14	−0.06	0.33	0.17	222	0.16	−0.02	0.34	0.08	219	0.12	−0.08	0.32	0.24	212	0.13	−0.05	0.31	0.15
MUFA																				
1st half of pregnancy	274	0.01	−0.20	0.21	0.95	263	−0.020	−0.200	0.170	0.86	258	−0.06	−0.24	0.13	0.54	249	−0.05	−0.23	0.13	0.62
3rd trimester	257	0.01	−0.16	0.19	0.88	247	0.05	−0.11	0.21	0.52	243	0.01	−0.16	0.18	0.93	234	0.02	−0.14	0.18	0.78
Combined 1st half of pregnancy and 3rd trimester	230	0.01	−0.28	0.29	0.97	222	0.04	−0.21	0.29	0.73	219	−0.07	−0.33	0.20	0.53	212	−0.03	−0.26	0.21	0.82
n-3 PUFA																				
1st half of pregnancy	274	−0.11	−1.33	1.11	0.86	263	−0.37	−1.55	0.81	0.54	258	−0.45	−1.44	0.53	0.37	249	−0.61	−1.67	0.45	0.26
3rd trimester	257	−0.52	−1.33	0.30	0.22	247	−0.39	−1.23	0.46	0.37	243	−0.37	−1.10	0.35	0.32	234	−0.25	−0.98	0.49	0.51
Combined 1st half of pregnancy and 3rd trimester	230	−1.14	−2.529	0.25	0.11	222	−0.94	−2.35	0.47	0.19	219	−1.06	−2.32	0.20	0.10	212	−0.89	−2.14	0.36	0.16
n-6 PUFA																				
1st half of pregnancy	274	−0.10	−0.50	0.29	0.62	263	−0.11	−0.48	0.26	0.55	258	−0.04	−0.42	0.33	0.82	249	−0.02	−0.38	0.34	0.92
3rd trimester	257	−0.12	−0.42	0.19	0.45	247	−0.05	−0.39	0.27	0.74	243	−0.03	−0.31	0.26	0.86	234	0.00	−0.31	0.30	0.99
Combined 1st half of pregnancy and 3rd trimester	230	−0.30	−0.74	0.14	0.19	222	−0.18	−0.62	0.26	0.43	219	−0.13	−0.55	0.28	0.53	212	−0.05	−0.48	0.39	0.84
Carbohydrates																				
1st half of pregnancy	274	−0.03	−0.10	0.05	0.50	263	−0.03	−0.10	0.04	0.42	258	0.002	−0.065	0.069	0.96	249	−0.01	−0.08	0.06	0.77
3rd trimester	257	−0.02	−0.08	0.04	0.54	247	−0.04	−0.10	0.03	0.25	243	−0.03	−0.09	0.04	0.47	234	−0.03	−0.10	0.03	0.31
Combined 1st half of pregnancy and 3rd trimester	230	−0.01	−0.11	0.08	0.75	222	−0.05	−0.14	0.04	0.28	219	−0.01	−0.10	0.09	0.87	212	−0.04	−0.12	0.05	0.42
Sucrose																				
1st half of pregnancy	274	0.01	−0.11	0.13	0.85	263	0.010	−0.120	0.140	0.87	258	0.09	−0.01	0.19	0.09	249	0.06	−0.06	0.17	0.31
3rd trimester	257	0.01	−0.08	0.09	0.85	247	−0.03	−0.14	0.07	0.55	243	−0.01	−0.10	0.08	0.78	234	−0.04	−0.15	0.06	0.40
Combined 1st half of pregnancy and 3rd trimester	230	0.05	−0.08	0.17	0.48	222	−0.03	−0.20	0.14	0.76	219	0.08	−0.05	0.20	0.22	212	−0.01	−0.17	0.15	0.91
Fiber																				
1st half of pregnancy	274	−0.03	−0.17	0.10	0.62	263	0.02	−0.12	0.15	0.82	258	−0.04	−0.15	0.07	0.52	249	−0.03	−0.14	0.09	0.66
3rd trimester	257	−0.11	−0.22	0.01	0.07	247	−0.04	−0.19	0.11	0.59	243	−0.06	−0.17	0.06	0.35	234	0.00	−0.14	0.13	0.95
Combined 1st half of pregnancy and 3rd trimester	230	−0.14	−0.29	0.01	0.07	222	−0.04	−0.22	0.13	0.62	219	−0.09	−0.24	0.05	0.21	212	−0.03	−0.19	0.14	0.77
Protein																				
1st half of pregnancy	274	0.05	−0.10	0.19	0.54	263	0.07	−0.08	0.22	0.36	257	−0.04	−0.17	0.88	0.55	249	−0.02	−0.16	0.12	0.75
3rd trimester	257	−0.04	−0.16	0.07	0.54	247	−0.03	−0.18	0.12	0.68	243	−0.04	−0.18	0.10	0.56	234	0.00	−0.15	0.15	0.98
Combined 1st half of pregnancy and 3rd trimester	230	0.02	−0.15	0.19	0.79	222	0.07	−0.16	0.30	0.54	219	0.01	−0.16	0.17	0.91	212	0.06	−0.13	0.28	0.61

ISO-BMI represents age- and sex- standardized BMI converted to adult scale and was calculated according to Saari et al. [23]. Model 1: adjusted for energy intake by energy density method (energy intake also added as a covariate in the multivariate model); Model 2: Model 1 + mother’s age, mother’s years of education, mother’s smoking, mother’s gestational diabetes status, mother’s intervention allocation in the RADIEL trial, prepregnancy BMI, offspring sex, offspring age during follow-up (months), sex- and gestational age-standardized birthweight, and offspring intake of the nutrient (E%). Bootstrap-type analyses. 1st half of pregnancy contains gestational weeks 5–18.

*SFA* saturated fatty acids, *MUFA* monounsaturated fatty acids, *PUFA* polyunsaturated fatty acids.

### Maternal macronutrient intake and offspring BFM and BF%

GDM status modified the association between first half of pregnancy n-3 PUFA intake and offspring BFM (*p* for interaction <0.001) and BF% (*p* for interaction = 0.001) at 5 years of age. In women with normal glucose metabolism during the index pregnancy, higher first half of pregnancy intake of n-3 PUFA was associated with lower offspring BFM and BF% at 5 years of age (Table 4). In women with GDM, however, higher first half of pregnancy intake of n-3 PUFA was associated with higher offspring BFM and BF% at 5 years of age. There were no differences in first half of pregnancy n-3 PUFA intakes between women with normal glucose tolerance and women with GDM (1.75 (0.39) vs. 1.8 (0.40), *p* = 0.70).

**Table 4:** Estimated change in offspring body fat mass and fat percentage associated with 1% increase in n-3 polyunsaturated fatty acid intake during pregnancy offset by concomitant isocaloric drop in other nutrients (total energy is held constant) stratified by gestational diabetes status, and *p* values for interaction with gestational diabetes status.

	Body fat mass (g)												Body fat percentage (%)											
	Model 1						Model 2						Model 1						Model 2					
	*n*	*p*_i_	*ß*	95% CI		*p*	*n*	*p*_i_	*ß*	95% CI		*p*	*p*_i_	*n*	*ß*	95% CI		*p*	*n*	*p*_i_	*ß*	95% CI		*p*
n-3 PUFA																								
1st half of pregnancy		0.011						<0.001					0.004							0.001				
GDM−	131		−0.73	−1.53	0.07	0.08	130		−0.90	−1.62	−0.18	0.01		131	−3.07	−5.95	−0.18	0.04	130.00		−3.45	−6.17	−0.72	0.01
GDM+	108		0.76	−0.09	1.60	0.08	103		0.94	0.14	1.75	0.02		108	2.70	−0.01	5.41	0.05	103		3.21	0.43	5.99	0.02
3rd trimester		0.70						0.59					0.84							0.89				
GDM−	129		−0.51	−1.14	0.12	0.11	128		−0.51	−1.14	0.12	0.11		129	−1.89	−4.26	0.48	0.12	128		−1.88	−4.18	0.43	0.11
GDM+	97		−0.33	−1.00	0.33	0.32	91		−0.32	−1.08	0.44	0.41		97	−1.51	−4.35	1.34	0.30	91		−1.62	−4.72	1.47	0.31
Total study population^a^	226		−0.43	−0.86	0.00	0.051	219		−0.41	−0.83	0.013	0.058		226	−1.71	−3.46	0.04	0.06	219		−1.69	−3.39	0.01	0.052

Model 1: adjusted for energy intake by energy density method (energy intake also added as a covariate in the multivariate model); Model 2: Model 1 + mother’s age, mother’s years of education, mother’s smoking, mother’s gestational diabetes status, mother’s intervention allocation in the RADIEL trial, prepregnancy BMI, offspring sex, offspring age during follow-up (months), sex- and gestational age-standardized birthweight, and offspring intake of the nutrient (E%). Bootstrap-type analyses. First half of pregnancy contains gestational weeks 5–18.

*PUFA* polyunsaturated fatty acid, *GDM−* no gestational diabetes, *GDM+* diagnosed gestational diabetes, *p*_i_ *p* for interaction with GDM.

^a^Total population presented in third trimester because no interaction was detected.

The association between maternal first half of pregnancy n-3 PUFA intake and offspring BF% pointed toward the same direction with and without adjustment for LTPA, both in women with normal glucose tolerance and in those with GDM. However, this association was not significant in those with GDM (Supplementary Table 1).

GDM did not modify the associations between third trimester maternal n-3 PUFA intake and BFM or BF%, and, in the total cohort, these associations were not statistically significant (Table 4). GDM did not modify any other associations between macronutrients and BFM and BF%. Prepregnancy BMI and breastfeeding did not modify any associations between macronutrients and BFM and BF% (all *p* for interaction >0.05).

Both higher third trimester SFA intake and higher combined SFA intake across pregnancy were associated with higher offspring BFM and BF% (in Models 1 and 2, Table 5).

**Table 5:** Estimated change in offspring body fat mass and fat percentage associated with 1% increase in specific macronutrient intake (except for fiber 1 g increase/1000 kcal) during pregnancy offset by concomitant isocaloric drop in other nutrients (total energy is held constant).

	Body fat mass (g)										Body fat percentage (%)									
	Model 1					Model 2					Model 1					Model 2				
	*n*	*ß*	95% CI		*p*	*n*	*ß*	95% CI		*p*	*n*	*ß*	95% CI		*p*	*n*	*ß*	95% CI		*p*
Total fat																				
1st half of pregnancy	240	0.02	−0.02	0.06	0.27	233	0.03	−0.01	0.06	0.17	240	0.09	−0.05	0.23	0.21	233	0.11	−0.02	0.24	0.106
3rd trimester	227	0.01	−0.03	0.05	0.56	219	0.02	−0.01	0.05	0.16	227	0.05	−0.07	0.18	0.41	219	0.07	−0.05	0.19	0.23
Combined 1st half of pregnancy and 3rd trimester	201	0.02	−0.03	0.07	0.46	195	0.04	−0.01	0.08	0.09	201	0.09	−0.08	0.26	0.30	195	0.14	−0.02	0.30	0.08
SFA																				
1st half of pregnancy	240	0.05	−0.03	0.13	0.25	233	0.06	−0.01	0.14	0.10	240	0.22	−0.07	0.51	0.14	233	0.27	−0.01	0.55	0.061
3rd trimester	227	0.06	0.00	0.12	0.07	219	0.07	0.01	0.12	0.014	227	0.25	0.03	0.47	0.029	219	0.26	0.07	0.45	0.008
Combined 1st half of pregnancy and 3rd trimester	201	0.08	−0.02	0.18	0.10	195	0.12	0.03	0.20	0.006	201	0.35	0.03	0.68	0.034	195	0.44	0.15	0.73	0.003
MUFA																				
1st half of pregnancy	240	0.05	−0.05	0.15	0.36	233	0.05	−0.04	0.15	0.29	240	0.18	−0.15	0.50	0.30	233	0.22	−0.11	0.55	0.19
3rd trimester	227	−0.01	−0.10	0.07	0.77	219	0.03	−0.05	0.10	0.48	227	−0.02	−0.32	0.28	0.91	219	0.09	−0.19	0.37	0.51
Combined 1st half of pregnancy and 3rd trimester	201	0.03	−0.11	0.16	0.68	195	0.08	−0.04	0.20	0.21	201	0.14	−0.29	0.58	0.52	195	0.31	−0.11	0.73	0.15
n-6 PUFA																				
1st half of pregnancy	240	0.01	−0.19	0.21	0.94	233	−0.001	−0.181	0.179	0.99	240	0.03	−0.70	0.64	0.93	233	−0.03	−0.68	0.61	0.92
3rd trimester	227	−0.08	−0.24	0.09	0.36	219	−0.05	−0.21	0.11	0.54	227	−0.21	−0.83	0.42	0.52	219	−0.20	−0.82	0.43	0.53
Combined 1st half of pregnancy and 3rd trimester	201	−0.11	−0.35	0.13	0.37	195	−0.07	−0.30	0.16	0.54	201	−0.31	−1.14	0.53	0.47	195	−0.18	−1.06	0.70	0.69
Carbohydrates																				
1st half of pregnancy	240	−0.02	−0.06	0.01	0.16	233	−0.03	−0.06	0.00	0.06	240	−0.07	−0.19	.0.5	0.23	233	−0.11	−0.23	0.00	0.058
3rd trimester	227	0.00	−0.04	0.03	0.80	219	−0.02	−0.05	0.02	0.274	227	−0.02	−0.15	0.10	0.72	219	−0.06	−0.19	0.07	0.38
Combined 1st half of pregnancy and 3rd trimester	201	−0.02	−0.06	0.03	0.50	195	−0.04	−0.09	−0.003	0.036	201	−0.06	−0.21	0.10	0.48	195	−0.15	−0.31	−0.001	0.050
Sucrose																				
1st half of pregnancy	240	0.01	−0.04	0.06	0.69	233	−0.01	−0.06	0.05	0.86	240	0.13	−0.06	0.32	0.20	233	0.05	−0.14	0.25	0.59
3rd trimester	227	0.03	−0.02	0.07	0.20	219	0.00	−0.05	0.05	0.99	227	0.12	−0.06	0.30	0.18	219	0.05	−0.15	0.25	0.63
Combined 1st half of pregnancy and 3rd trimester	201	0.05	−0.02	0.11	0.16	195	−0.02	−0.10	0.07	0.67	201	0.24	−0.02	0.50	0.07	195	0.01	−0.27	0.30	0.92
Fiber																				
1st half of pregnancy	240	−0.05	−0.10	0.01	0.12	233	−0.02	−0.08	0.04	0.43	240	−0.16	−0.37	0.04	0.12	233	−0.12	−0.33	0.09	0.28
3rd trimester	227	−0.04	−0.09	0.01	0.09	219	−0.01	−0.08	0.05	0.68	227	−0.14	−0.34	0.05	0.16	219	−0.03	−0.25	0.19	0.77
Combined 1st half of pregnancy and 3rd trimester	201	−0.08	−0.15	−0.01	0.02	195	−0.04	−0.12	0.04	0.36	201	−0.27	−0.53	−0.01	0.043	195	−0.13	−0.41	0.15	0.36
Protein																				
1st half of pregnancy	240	0.02	−0.05	0.09	0.52	233	0.03	−0.04	0.10	0.41	240	−0.01	−0.25	0.23	0.95	233	0.05	−0.19	0.29	0.69
3rd trimester	227	−0.02	−0.08	0.05	0.57	219	−0.01	−0.08	0.07	0.84	227	−0.11	−0.37	0.14	0.39	219	−0.07	−0.30	0.19	0.58
Combined 1st half of pregnancy and 3rd trimester	201	0.01	−0.08	0.09	0.83	195	0.04	−0.06	0.16	0.45	201	−0.07	−0.37	0.24	0.66	195	0.07	−0.28	0.42	0.69

Model 1: adjusted for energy intake by energy density method (energy intake also added as a covariate in the multivariate model); Model 2: Model 1 + mother’s age, mother’s years of education, mother’s smoking, mother’s gestational diabetes status, mother’s intervention allocation in the RADIEL trial, prepregnancy BMI, offspring sex, offspring age during follow-up (months), sex- and gestational age-standardized birthweight, and offspring intake of the nutrient (E%). Bootstrap-type analyses. First half of pregnancy contains gestational weeks 5–18. Results of n-3 PUFA are presented stratified by gestational diabetes because of a modifying effect of gestational diabetes on some associations.

*SFA* saturated fatty acid, *MUFA* monounsaturated fatty acid, *PUFA* polyunsaturated fatty acid.

Higher carbohydrate (CHO) intake across pregnancy was associated with lower offspring BFM and borderline associated with lower BF% (in Model 2). No other associations between maternal macronutrient intakes and offspring BFM and BF% were observed.

### Sensitivity analyses

Because maternal n-3 PUFA, SFA, and CHO intakes during pregnancy were associated with offspring ISO-BMI, BFM, and BF%, we also examined the associations between maternal n-3 PUFA, SFA, and CHO intakes at 12 months and 5 years after delivery and offspring anthropometric indicators. However, no associations with offspring ISO-BMI, BFM, or BF% at 12 months or 5 years were detected (Supplementary Table 3). No quadratic associations between maternal macronutrient intakes and offspring BFM or BF% were found.

## Discussion

We found that GDM modified the associations between maternal intake of n-3 PUFAs in the first half of pregnancy and offspring BFM and BF% at 5 years of age. Maternal intake of SFA during pregnancy was positively associated with offspring ISO-BMI, BFM, and BF%. In contrast, maternal CHO intake across pregnancy was negatively associated with offspring BFM and BF%.

Only a few previous human studies have examined associations between maternal macronutrient intakes during pregnancy and offspring adiposity after infancy [8, 16–19]. Among the strengths of our study are the collection of detailed maternal nutrient intake data at four timepoints, during and after pregnancy, and the inclusion of data on the offspring’s own nutrient intake. These enabled us to assess the role of timing of maternal macronutrient intake during pregnancy and the food environment of the offspring after birth in the development of offspring adiposity. No associations between maternal macronutrient intakes after pregnancy and offspring anthropometric indictors were found, and our analyses were adjusted for the child’s own nutrient intake at 5 years. Hence, it is unlikely that our findings result from confounding by maternal or child diet composition after delivery.

Compared to the results of the present study, previous cohort studies have reported both congruent [17, 18] and discrepant [19] findings on the association of maternal n-3 PUFA intake during pregnancy and offspring adiposity. Differences in the impact of maternal n-3 PUFA intakes in healthy women vs. women with GDM in the first half of pregnancy on offspring body composition could explain some of these inconsistencies, as earlier studies have examined average nutrient intakes across the entire pregnancy. More recently, Brei et al. [8] showed that higher late pregnancy n-3 PUFA intake is associated with lower birthweight and newborn fat mass in the offspring, although these associations did not persist until 1 year of age. Donahue et al. (*n* = 1120), however, found an association between higher maternal n-3 PUFA intake and lower sum of subscapular and tricep skinfold thicknesses in children at 3 years of age [18], conforming to our results in women without GDM. n-3 PUFAs block adipose tissue development, leading to lower fat mass [24], which could possibly contribute to the association of late pregnancy maternal PUFA intake with reduced offspring adiposity, although this finding did not reach significance in our study. In a mouse model, increased maternal n-3 fatty acid intake (reduced n-6/n-3 PUFA ratio) was shown to lead to lower body fat and traits indicative of an obesity-resistant phenotype in the offspring [24]. However, although cohort studies have repeatedly found inverse associations between maternal n-3 PUFA intake during pregnancy and offspring adiposity [18, 25], these have not been replicated in clinical trials testing n-3 PUFA supplementation in pregnant women [26].

Long-chain PUFAs are essential for normal fetal growth and development. Since placental and fetal capacity to synthesize them is low, the fetus depends on the maternal supply of PUFAs [27]. Earlier studies have suggested derangements in the placental transfer and/or feto-placental metabolism of PUFAs in late pregnancy in women with GDM or obesity [28–32], which could contribute to the lack of benefit on offspring adiposity from increased late pregnancy n-3 PUFA intake in our study population. Similar data on the first half of pregnancy is lacking and the flux of lipids to the fetus is considerably lower at <20 weeks gestation than at term [28]. However, GDM is characterized by various maternal metabolic abnormalities (e.g., elevated glucose and insulin) already in early pregnancy [33, 34], and early changes in placental metabolism and growth in response to disturbed maternal metabolism are increasingly recognized as potential factors in the pathogenesis of fetal adiposity [35–38]. Considering our observation of increased offspring adiposity in women with GDM with high first half of pregnancy n-3 PUFA intake, it is possible that placental n-3 PUFA metabolism is altered in GDM already in the first half of pregnancy.

Current evidence regarding the impact of maternal SFA intake on offspring body composition is inconsistent. In a large Finnish birth cohort study by Hakola et al. (*n* = 3807) [17], maternal cumulative SFA intake during pregnancy was not associated with offspring BMI or overweight at 2–7 years [17]. Likewise, Brei et al. (*n* = 208) [8] did not find associations between SFA intake in early or late pregnancy and offspring weight, BMI, BFM, or subcutaneous or peritoneal fat area at birth or at the age of 1, 3, or 5 years. The participants in these studies were leaner and the proportion of mothers with GDM was null [17] or considerably lower [8], compared to the mothers of the present study. This may have contributed to the discrepancy in results, especially considering the differences in maternal, placental, and fetal lipid metabolism that characterize GDM/obese vs. non-GDM/non-obese pregnancies [28, 39]. Murrin et al. [16], on the other hand, discovered a positive association between maternal SFA intake in the first trimester and offspring weight at 5 years of age in 585 mother–child dyads. This result, if weight is interpreted as an indication of adiposity, is in line with our findings concerning third trimester SFA intake and combined SFA intake across gestation. It is possible that our smaller sample size may have diluted the association between the first half of pregnancy intake and offspring anthropometric indicators. Unfortunately, Murrin et al. did not report maternal background characteristics, such as BMI or GDM status, disallowing detailed comparisons [16]. Data from animal studies are consistent with our findings, since high maternal SFA intake has been shown to result in increased neonatal body mass [40], later adiposity [41], beta-cell loss and glucose intolerance [42], as well as impaired appetite regulation [43], in the offspring.

In the present study, maternal SFA intake during late pregnancy, as opposed to the first half of pregnancy, displayed a more pronounced association with offspring anthropometric indicators. This is logical since the last trimester of pregnancy is characterized by the most rapid fetal growth and fat accretion, which is further promoted by maternal obesity and/or diabetes which enhance the availability of lipids as a feto-placental fuel and trigger fetal hyperinsulinemia [28]. Nearly half of our cohort was composed of women with GDM, in whom maternal late pregnancy free fatty acid, glycerol and triacylglycerol concentrations have been shown to correlate with cord lipid concentrations, newborn size, and adiposity [44, 45]. An SFA-rich diet could also contribute to poorer maternal glycemic control via worsening of late-pregnancy insulin resistance [46], especially in our metabolically compromised study population. It is also possible that excess SFA could suppress the activity of specific enzymes (Δ5 and Δ6) necessary for the synthesis of metabolically important long-chain PUFAs in maternal and fetal tissues [47].

Brei et al. [8] found, in 186 mother–child dyads, that an incremental increase in maternal fat and protein intake at the expense of CHO in late but not in early pregnancy was associated with lower offspring BFM up to 5 years. This is in contrast to our study in which higher maternal CHO intake was associated with lower offspring BFM and BF% at 5 years of age. However, the average CHO intake was ~7 E% units higher in the study population of Brei et al. as compared to our study population, which could explain the discrepancy in our results. The finding that only cumulative CHO intake across pregnancy was associated with BFM and BF% probably attributes to the higher accuracy of measurement achieved with 6 days rather than 3 days of food records.

We did not find an association between maternal macronutrient intake and newborn relative birthweight, in agreement with some studies [41] but in contradiction to others [48]. Our results may reflect the fact that birthweight is not a measure of adiposity. It is possible that the children with the highest BFM/BF% values already had higher adiposity at birth [49], but it is also plausible that fetal programming predisposed them to increased fat accumulation after the neonatal period. The latter speculation is supported by a meta-analysis of studies in rodents, which concluded that maternal high-fat (mostly saturated) intake did not affect offspring birthweight but increased weaning weight, final bodyweight, and adiposity [41]. This is in agreement with our results showing no association between maternal SFA intake and offspring birthweight, but positive associations between maternal SFA intake and offspring BFM and BF% at 5 years of age. The possible cause–effect relationships between maternal SFA intake and offspring metabolism are myriad and remain to be elucidated, with potential mechanisms ranging from adverse impacts of SFA on feto-placental mitochondrial metabolism to alterations of maternal/fetal gut microbiota [50–52].

A strength of our study is the availability of direct measurement of offspring adiposity as BFM and BF%, instead of relying only on bodyweight, BMI, or WHtR [53]. We were able to adjust for a wide range of variables, in order to minimize residual confounding. Since all the women who participated in the present study were either obese or had a history of GDM, and consequently their fetuses were exposed to an altered intrauterine environment, the results cannot be generalized to metabolically healthy pregnancies. Our relatively small sample size is another limitation, which may have attenuated some of the observed associations between maternal macronutrient intakes and offspring anthropometric indicators. Due to lack of data on newborn BFM, BF%, or metabolic parameters (e.g., insulin or C-peptide levels), we were not able to assess whether associations between specific macronutrient intakes and offspring adiposity already existed in infancy. Similarly, due to lack of data, we could not adjust for maternal macronutrient intake during lactation as a possible confounder.

In metabolically burdened women, associations between the first half of pregnancy n-3 PUFA intake and offspring BFM and BF% at 5 years of age were modified by GDM. In the first half of pregnancy, higher n-3 PUFA intake was associated with lower offspring BFM and BF% in women with normal glucose tolerance during pregnancy and higher offspring BFM and BF% in women with GDM. Higher intake of SFA during pregnancy was associated with higher ISO-BMI, BFM, and BF% in the offspring at 5 years of age, whereas higher intake of CHOs was associated with lower offspring BFM and BF%. This study introduces novel evidence of possible fetal programming by maternal macronutrient intake in metabolically burdened women who constitute a growing proportion of obstetric populations worldwide. Taken together, our findings add to the accumulating evidence on the role of maternal macronutrient intake in offspring metabolic programming and suggest that its effects may vary between normoglycemic women and women with GDM and depend on the timing of feto-placental exposure.

## Supplementary information

Supplemental table 1

Supplemental table 2

Supplemental table 3

## Data Availability

The dataset supporting the conclusions of this article is available upon a reasonable request from the authors.

## Code availability

The code supporting the conclusions of this article is available upon a reasonable request from the authors.
