# Supplementary material for: Macronutrient intake during pregnancy in women with a history of obesity or gestational diabetes and offspring adiposity at 5 years of age
Source: Int J Obes (Lond). 2021 Feb 8;45(5):1030–43. doi: 10.1038/s41366-021-00762-0 (PMC8081655; doi:10.1038/s41366-021-00762-0)
Supplement: Supplementary file 1 — Supplemental table 1 [file 41366_2021_762_MOESM1_ESM.docx]

| **Supplemental table 1. Estimated change in offspring body fat percentage and fat mass associated with 1% increase in specific macronutrient intake (except for fiber 1g increase/ 1000kcal) during pregnancy offset by concomitant isocaloric drop in other nutrients (total energy is held constant) including (A) leisure-time physical activity (min/wk) or (B) duration of breastfeeding (<4 months / ≥4 months) as a covariate.** | | | | | | | | | | | |
| --- | --- | --- | --- | --- | --- | --- | --- | --- | --- | --- | --- |
|  | **(A) Additional adjustment for leisure-time physical activity** | | | | |  | **(B) Additional adjustment for duration of breastfeeding** | | | | |
|  | n | ß | 95% CI | | p |  | n | ß | 95% CI | | p |
| **FAT** |  |  |  |  |  |  |  |  |  |  |  |
| 1st half of pregnancy | 202 | 0.12 | -0.02 | 0.26 | 0.09 |  | 211 | 0.12 | -0.02 | 0.25 | 0.09 |
| 3 trim | 205 | 0.07 | -0.05 | 0.19 | 0.25 |  | 199 | 0.07 | -0.04 | 0.19 | 0.22 |
| Combined 1st half of pregnancy and 3rd trimester | 158 | 0.15 | -0.03 | 0.32 | 0.11 |  | 177 | 0.15 | 0.00 | 0.30 | 0.05 |
| **SFA** |  |  |  |  |  |  |  |  |  |  |  |
| 1st half of pregnancy | 202 | 0.37 | 0.08 | 0.65 | 0.01 |  | 211 | 0.23 | -0.07 | 0.54 | 0.13 |
| 3 trim | 205 | 0.26 | 0.06 | 0.46 | 0.01 |  | 199 | 0.21 | 0.00 | 0.43 | 0.05 |
| Combined 1st half of pregnancy and 3rd trimester | 158 | 0.51 | 0.19 | 0.82 | 0.00 |  | 177 | 0.35 | 0.05 | 0.65 | 0.02 |
| **MUFA** |  |  |  |  |  |  |  |  |  |  |  |
| 1st half of pregnancy | 202 | 0.21 | -0.14 | 0.55 | 0.24 |  | 211 | 0.27 | -0.07 | 0.62 | 0.12 |
| 3 trim | 205 | 0.10 | -0.20 | 0.40 | 0.51 |  | 199 | 0.11 | -0.18 | 0.40 | 0.47 |
| Combined 1st half of pregnancy and 3rd trimester | 158 | 0.22 | -0.25 | 0.70 | 0.36 |  | 177 | 0.15 | -0.04 | 0.34 | 0.13 |
| **n-3 PUFA** |  |  |  |  |  |  |  |  |  |  |  |
| 1st half of pregnancy | 202 | -1.12 | -3.19 | 0.94 | 0.29 |  |  |  |  |  |  |
| GDM- | 114 | -3.89 | -6.88 | -0.91 | 0.01 |  | 121 | -3.80 | -6.73 | -0.88 | 0.01 |
| GDM+ | 88 | 2.17 | -0.86 | 5.21 | 0.16 |  | 90 | 3.54 | 0.19 | 6.88 | 0.04 |
| 3 trim | 205 | -1.82 | -3.55 | -0.09 | 0.04 |  | 199 | -0.88 | -2.66 | 0.90 | 0.33 |
| GDM- | 123 | -1.86 | -4.26 | 0.55 | 0.13 |  | 119 | -0.91 | -3.07 | 1.26 | 0.41 |
| GDM+ | 82 | -1.49 | -4.93 | 1.96 | 0.40 |  | 80 | -0.83 | -4.55 | 2.89 | 0.66 |
| **n-6 PUFA** |  |  |  |  |  |  |  |  |  |  |  |
| 1st half of pregnancy | 202 | -0.18 | -0.86 | 0.50 | 0.60 |  | 211 | 0.02 | -0.65 | 0.70 | 0.95 |
| 3 trim | 205 | -0.24 | -0.90 | 0.43 | 0.49 |  | 199 | -0.08 | -0.73 | 0.57 | 0.82 |
| Combined 1st half of pregnancy and 3rd trimester | 158 | -0.40 | -1.36 | 0.56 | 0.42 |  | 177 | -0.01 | -0.90 | 0.87 | 0.98 |
| **CHO** |  |  |  |  |  |  |  |  |  |  |  |
| 1st half of pregnancy | 202 | -0.12 | -0.24 | 0.01 | 0.07 |  | 211 | -0.10 | -0.22 | 0.02 | 0.12 |
| 3 trim | 205 | -0.07 | -0.20 | 0.06 | 0.29 |  | 199 | -0.06 | -0.19 | 0.07 | 0.36 |
| Combined 1st half of pregnancy and 3rd trimester | 158 | -18.53 | -35.19 | -1.87 | 0.03 |  | 177 | -0.03 | -0.06 | 0.00 | 0.06 |
| **Sucrose** |  |  |  |  |  |  |  |  |  |  |  |
| 1st half of pregnancy | 202 | 0.03 | -0.18 | 0.25 | 0.75 |  | 211 | 0.01 | -0.03 | 0.05 | 0.71 |
| 3 trim | 205 | 0.02 | -0.19 | 0.23 | 0.87 |  | 199 | 0.02 | -0.21 | 0.24 | 0.87 |
| Combined 1st half of pregnancy and 3rd trimester | 158 | -0.09 | -0.46 | 0.28 | 0.64 |  | 177 | -0.08 | -0.38 | 0.22 | 0.59 |
| **Fiber** |  |  |  |  |  |  |  |  |  |  |  |
| 1st half of pregnancy | 202 | -0.12 | -0.35 | 0.11 | 0.32 |  | 211 | -0.11 | -0.33 | 0.11 | 0.33 |
| 3 trim | 205 | -0.02 | -0.26 | 0.23 | 0.89 |  | 199 | 0.00 | -0.23 | 0.23 | 0.98 |
| Combined 1st half of pregnancy and 3rd trimester | 158 | -0.07 | -0.44 | 0.29 | 0.69 |  | 177 | -0.10 | -0.39 | 0.19 | 0.50 |
| **Protein** |  |  |  |  |  |  |  |  |  |  |  |
| 1st half of pregnancy | 202 | 0.03 | -0.24 | 0.30 | 0.84 |  | 211 | -0.03 | -0.30 | 0.23 | 0.80 |
| 3 trim | 205 | -0.02 | -0.29 | 0.26 | 0.90 |  | 199 | -0.09 | -0.38 | 0.21 | 0.57 |
| Combined 1st half of pregnancy and 3rd trimester | 158 | 0.14 | -0.29 | 0.57 | 0.53 |  | 177 | 0.03 | -0.36 | 0.42 | 0.89 |
| SFA, saturated fatty acids; MUFA, monounsaturated fatty acids; PUFA, polyunsaturated fatty acid; GDM-, no gestational diabetes; GDM+, diagnosed gestational diabetes; CHO, carbohydrates. Adjusted for energy intake (energy density method), mother's age, mother's years of education, mother's smoking, maternal pre-pregnancy BMI, mother's GDM status, mother's intervention allocation in the RADIEL trial, gestational age standardized birth weight, offspring sex, offspring age during 5 year follow-up visit, offspring intake of the nutrient (E%), and (A) maternal leisure-time physical activity during each time point, or (B) duration of breastfeeding. 1st half of pregnancy contains gestational weeks from 5 to 18. For n-3 PUFA, the estimates are given separately in women with normal glucose metabolism during pregnancy and women with GDM because GDM significantly modified the association between early pregnancy n-3 PUFA intake and offspring BF% at 5 years of age. | | | | | | | | | | | |
