# Supplementary material for: Macronutrient intake during pregnancy in women with a history of obesity or gestational diabetes and offspring adiposity at 5 years of age
Source: Int J Obes (Lond). 2021 Feb 8;45(5):1030–43. doi: 10.1038/s41366-021-00762-0 (PMC8081655; doi:10.1038/s41366-021-00762-0)
Supplement: Supplementary file 2 — Supplemental table 2 [file 41366_2021_762_MOESM2_ESM.docx]

| Supplemental Table 2. Estimated change in sex- and gestational age– standardized birthweight (SD) associated with a 1% increase in specific macronutrient intake (except for fiber 1g increase/1000kcal) during pregnancy offset by concomitant isocaloric drop in other nutrients (total energy is held constant). | | | | | |
| --- | --- | --- | --- | --- | --- |
|  | n | ß | 95% CI | | p |
| Total fat |  |  |  |  |  |
| 1st half of pregnancy | 272 | 0.01 | -0.01 | 0.04 | 0.33 |
| 3rd trimester | 253 | 0.00 | -0.02 | 0.02 | 0.81 |
| Combined 1st half of pregnancy and 3rd trimester | 228 | 0.00 | -0.02 | 0.03 | 0.90 |
| SFA |  |  |  |  |  |
| 1st half of pregnancy | 272 | 0.01 | -0.03 | 0.06 | 0.53 |
| 3rd trimester | 253 | -0.01 | -0.05 | 0.03 | 0.51 |
| Combined 1st half of pregnancy and 3rd trimester | 228 | -0.01 | -0.07 | 0.04 | 0.61 |
| MUFA |  |  |  |  |  |
| 1st half of pregnancy | 272 | 0.02 | -0.04 | 0.08 | 0.50 |
| 3rd trimester | 253 | 0.00 | -0.05 | 0.05 | 0.96 |
| Combined 1st half of pregnancy and 3rd trimester | 228 | 0.01 | -0.06 | 0.08 | 0.78 |
| n-3 PUFA |  |  |  |  |  |
| 1st half of pregnancy | 272 | 0.23 | -0.07 | 0.53 | 0.13 |
| 3rd trimester | 253 | 0.07 | -0.18 | 0.33 | 0.57 |
| Combined 1st half of pregnancy and 3rd trimester | 228 | 0.32 | -0.09 | 0.74 | 0.13 |
| n-6 PUFA |  |  |  |  |  |
| 1st half of pregnancy | 272 | 0.05 | -0.04 | 0.15 | 0.27 |
| 3rd trimester | 253 | 0.01 | -0.08 | 0.09 | 0.87 |
| Combined 1st half of pregnancy and 3rd trimester | 228 | 0.06 | -0.07 | 0.18 | 0.36 |
| Carbohydrates |  |  |  |  |  |
| 1st half of pregnancy | 272 | 0.00 | -0.03 | 0.02 | 0.76 |
| 3rd trimester | 253 | 0.01 | -0.01 | 0.02 | 0.55 |
| Combined 1st half of pregnancy and 3rd trimester | 228 | 0.00 | 0.00 | 0.01 | 0.52 |
| Sucrose |  |  |  |  |  |
| 1st half of pregnancy | 272 | -0.01 | -0.01 | 0.00 | 0.13 |
| 3rd trimester | 253 | 0.01 | -0.02 | 0.04 | 0.44 |
| Combined 1st half of pregnancy and 3rd trimester | 228 | -0.02 | -0.07 | 0.03 | 0.41 |
| Fiber |  |  |  |  |  |
| 1st half of pregnancy | 272 | 0.01 | -0.02 | 0.04 | 0.58 |
| 3rd trimester | 253 | 0.00 | -0.03 | 0.04 | 0.88 |
| Combined 1st half of pregnancy and 3rd trimester | 228 | 0.01 | -0.03 | 0.05 | 0.65 |
| Protein |  |  |  |  |  |
| 1st half of pregnancy | 272 | -0.03 | -0.07 | 0.01 | 0.17 |
| 3rd trimester | 253 | -0.02 | -0.06 | 0.02 | 0.40 |
| Combined 1st half of pregnancy and 3rd trimester | 228 | -0.04 | -0.10 | 0.01 | 0.15 |
| SFA, saturated fatty acids; MUFA, monounsaturated fatty acids; PUFA, polyunsaturated fatty acid. Adjusted for energy intake (energy density method), mother's age, years of education, smoking, pre-pregnancy BMI, GDM status, intervention allocation, and offspring sex. Bootstrap-type analyses. 1st half of pregnancy contains gestational weeks 5–18. | | | | | |
