# Supplementary material for: Macronutrient intake during pregnancy in women with a history of obesity or gestational diabetes and offspring adiposity at 5 years of age
Source: Int J Obes (Lond). 2021 Feb 8;45(5):1030–43. doi: 10.1038/s41366-021-00762-0 (PMC8081655; doi:10.1038/s41366-021-00762-0)
Supplement: Supplementary file 3 — Supplemental table 3 [file 41366_2021_762_MOESM3_ESM.docx]

| Supplementary Table 3. Estimated change in offspring ISO-BMI, body fat mass, and body fat percentage associated with a 1% isocaloric increase in specific macronutrient intake at 12 months and 5 years after pregnancy offset by concomitant drop in other nutrients (total energy is held constant). | | | | | | | | | | | | | | |
| --- | --- | --- | --- | --- | --- | --- | --- | --- | --- | --- | --- | --- | --- | --- |
|  | ISO-BMI | | | |  | Body fat mass (g) | | | |  | Body fat percentage (%) | | | |
| SFA | β | 95% CI | | p |  | β | 95% CI | | p |  | β | 95% CI | | p |
| 12 months pp | 0.10 | -0.04 | 0.23 | 0.15 |  | 0.04 | -0.01 | 0.1 | 0.12 |  | 0.14 | -0.09 | 0.36 | 0.23 |
| 5 years pp | 0.04 | -0.11 | 0.18 | 0.64 |  | 0.04 | -0.03 | 0.11 | 0.31 |  | 0.19 | -0.07 | 0.45 | 0.19 |
| n-3 PUFA |  |  |  |  |  |  |  |  |  |  |  |  |  |  |
| 12 months pp | - |  |  |  |  | -0.11 | -0.5 | 0.29 | 0.6 |  | -0.16 | -1.69 | 1.38 | 0.84 |
| 5 years pp | - |  |  |  |  | 0.01 | -0.12 | 0.15 | 0.84 |  | 0.04 | -0.49 | 0.59 | 0.88 |
| Carbohydrate |  |  |  |  |  |  |  |  |  |  |  |  |  |  |
| 12 months pp | - |  |  |  |  | 0 | -0.02 | 0.02 | 0.97 |  | 0 | -0.1 | 0.1 | 0.99 |
| 5 years pp | - |  |  |  |  | -0.01 | -0.04 | 0.01 | 0.37 |  | -0.06 | -0.16 | 0.05 | 0.28 |
| ISO-BMI, sex-specific BMI-for-age [2]; SFA, saturated fatty acids; PUFA, polyunsaturated fatty acid. Analyses adjusted for mother's energy intake, age during pregnancy, educational attainment (years), smoking in the 1st trimester of pregnancy (yes/no), gestational diabetes status (yes/no), intervention allocation (control/intervention during the main trial), maternal BMI in the 1st trimester, offspring sex, offspring age, and offspring’s relative birthweight (z score) [1]. Linear regression analyses using bootstrap method. | | | | | | | | | | | | | | |

References:

(1) Pihkala J, Hakala T, Voutilainen P, Raivio K. [Characteristic of recent fetal growth curves in Finland]. Duodecim 1989;105(18):1540-1546.

(2) Saari A, Sankilampi U, Hannila M, Kiviniemi V, Kesseli K, Dunkel L. New Finnish growth references for children and adolescents aged 0 to 20 years: Length/height-for-age, weight-for-length/height, and body mass index-for-age. Ann Med 2011 May;43(3):235-248.
